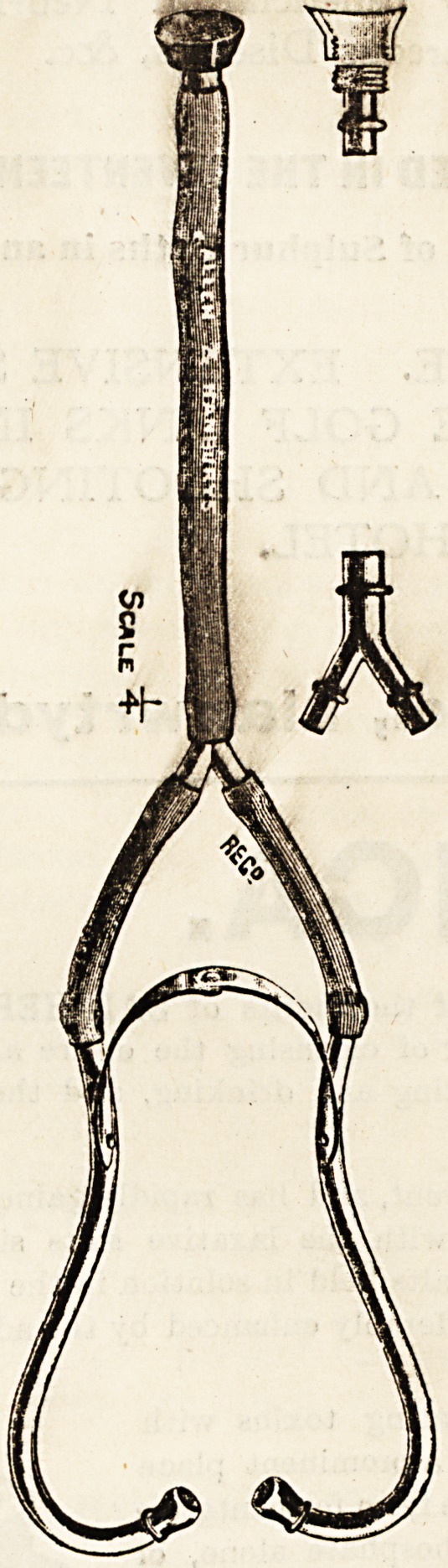# New Appliances & Things Medical

**Published:** 1911-08-19

**Authors:** 


### NEW APPLIANCES & THINGS MEDICAL.

### A GENERAL PRACTITIONER'S STETHOSCOPE.

Dr. T. C. Blackwell, of Oakshott, Surrey, writes to us as follows: Messrs. Allen and Hanbury have made for me the stethoscope shown in the diagrams.

The improvements claimed are that it is mathematically and physically correct throughout. The combined sectional areas of the two smaller tubes are equal to the sectional areas of the larger tube. There is no widening or narrowing of the lumen of the tube from chest-piece to ear, and therefore nothing to interfere with the sound waves. This is achieved by the following methods. The worm on the chest-piece is external; the diameter of the rubber tube is the same as that of the proximal end of the chest-piece; the latter has a knife edge so that the constriction which ordinarily occurs immediately beyond the attachment of a rubber tube to a rigid tube does not take place; the three orifices of the "Y" tube have also knife edges. There is no lessening of the calibre at the junction of the tube and earpiece. The opening of the latter is slightly larger than that of the tube, so that when placed in the ear there is no choking. The advantages of the instrument are that all exaggeration of the sound, and also any accentuation of a component of the sound beyond another are avoided, and the exact sound produced is conveyed to the ear. Moreover, adventitious sounds are excluded with great ease. This is particularly noticeable in listening to the breath sounds. The convenience of the single tube is very great. If the test recommended by Hutchison and Rainy in their "Clinical Methods" be applied, it will be found that the intensity of the sound—the ticking of a watch—is at least double that of an ordinary binaural instrument.

I have to express my indebtedness to Mr. Lewis, of Messrs. Allen and Hanbury, for his courtesy and his interest in the designing and completing of the instrument.

## Figures and Tables

**Figure f1:**